# A Retrospective Analysis of Ventilatory Strategy Comparing Non-invasive Ventilation (NIV) With Invasive Ventilation in Patients Admitted With Severe COVID-19 Pneumonia

**DOI:** 10.7759/cureus.34249

**Published:** 2023-01-26

**Authors:** Madhu Srinivasaiah, Manu M Krishnappa Gowda Varma, Nandini M G, Chaitra V, Harshitha Gulur, Harshitha V

**Affiliations:** 1 Anaesthesiology, Dr. Chandramma Dayananda Sagar Institute of Medical Education and Research, Bengaluru, IND; 2 Critical Care Medicine, Dr. Chandramma Dayananda Sagar Institute of Medical Education and Research, Bengaluru, IND; 3 Internal Medicine, Dr. Chandramma Dayananda Sagar Institute of Medical Education and Research, Bengaluru, IND

**Keywords:** ards, invasive positive pressure ventilation, niv, icu, covid-19

## Abstract

Background

The second wave of the COVID-19 pandemic in India saw a sudden upsurge of critically ill patients getting admitted to the ICU. The guidance for respiratory support was unclear in the early phase. But later reports showed lower mortality with non-invasive ventilation (NIV) than with intubation. The aim of this study was to assess the end result of initial methods of ventilation in COVID-19 patients.

Methodology

Patients admitted to ICU with COVID-19 were categorized as group 1 (IPPV-intubated within 24 hrs of admission), group 2 (NIV -NIV only), group 3 (NIV+ IPPV-intubated after 24 hrs), and group 4 (NRBM - Non-Rebreathing Mask only). All causes in the hospital or 30-day mortality, length of stay in ICU, and incidence of pneumothorax were compared between groups. Logistic regression analysis was done to determine the odds of mortality.

Results

The overall mortality rate among patients admitted to tertiary care centers was 15% and the rate among patients in ICU was 54.07%. Patients in group 1 and group 3 had significantly high mortality rates of 90.47% and 93.75%, respectively, as compared to 51.28% in group 2 patients. The odds of mortality were high in group 3 (OR 29.57, 95% CI 4.51 and 193.52) and group 1 (OR 8.01, 95% CI 1.35 and 47.48).

Conclusion

In a resource-limited setting, the use of NIV is associated with higher survival in COVID-19 patients. The prognosis of patients who are intubated early or after a trial of NIV is the same with increased odds of mortality.

## Introduction

On March 11, 2020, COVID-19 was declared a global pandemic. In India, the first case was identified in January 2020 [1]. The first wave in India was prolonged and extended from March 2020 to February 2021 [2]. The peak cases per million in the first wave was around 96,760 new cases/day during mid-September and declined to 11,436 cases/day by the end of December 2020. However, by the end of March 2021, India experienced an unexpected rise in confirmed COVID-19 cases and at the peak of second wave the daily new cases were 414,433 [3]. This sudden upsurge in cases was associated with a high volume of critically ill patients getting admitted to the ICU.

The respiratory failure caused by COVID-19 was described in terms of acute respiratory distress syndrome (ARDS) in various case series and initial reports. ARDS is characterized by diffuse inflammation of lung parenchyma leading to increased capillary permeability and hence decreased gas exchange leading to type I respiratory failure [4]. The use of non-invasive ventilation (NIV) in the management of respiratory failure due to ARDS is controversial [5]. The guidance for respiratory support in COVID-19 was in favor of early intubation than NIV in the earlier phase of pandemic with risk of aerosolization being the major determinant factor for recommendation [6]. However, as the pandemic progressed, later reports showed lower mortality with the use of NIV and high-flow nasal oxygen (HFNC) than with intubation [7-11].

The reported mortality of patients admitted in ICU during first wave across globe had ranged from 26% to 69% in various case series published around mid-2020 [12-14]. In India, the mortality of patients admitted in ICU during first wave was around 13.4% [15]. In mid-March 2021, during the beginning of second wave, ICUs in India were overwhelmed with increased number of critically ill patients getting admitted. Ventilatory strategy in resource limited settings was a huge challenge. This retrospective study in a tertiary care center was designed to analyze the outcome of initial method of ventilation in patients with COVID-19 respiratory failure during second wave in India.

## Materials and methods

This study was a retrospective cohort study, including all patients admitted to the critical care unit at our institute with COVID-19 (SAR-CoV-2). Patients above the age of 18 years and those admitted between March 2021 and August 2021 were included in this study. COVID-19 diagnoses confirmed by RT-PCR or CT scan were included. The study data collection was started after obtaining the Institutional scientific committee and Institutional Ethics committee (IEC) approval. As the study was a retrospective study, a waiver for informed consent was obtained from IEC. The study was registered in the clinical trial registry.

The files of patients admitted to the ICU were obtained from the Medical Record Department as per existing protocol. The data from files were entered into Case Report Form (CRF)/Data Collection Form in excel format capturing the essential data required.

Demographic data, duration of symptoms, co-morbidities, baseline SpO_^2^_, and SpO_2_/FiO_2_ ratio were collected from the patient’s initial assessment form in the emergency department. Patients were categorized in the emergency department to moderate and severe illness based on the algorithm issued by AIIMS/ICMR - COVID-19 national task force dated April 22, 2021. Severe cases were admitted to ICU and treated as per guidelines. COVID-19-specific treatments such as steroids, anticoagulants, antiviral, and immunomodulators were administered as per the treatment guidelines. Daily progress sheets and ventilation charts were screened for documentation of the ventilation strategy adopted. NIV was defined as if the physician had documented or had an order written in the daily progress sheet. Invasive positive pressure ventilation (IPPV) was defined as documented evidence of intubation or some advice in the case sheet. The mode of ventilation used was categorized as NRBM, NIV, or IPPV based on documentation in the daily progress sheet. In order to consider patients with two or more modalities of ventilation used in a 24hr period, they were categorized into the highest form of ventilation adopted, the order being IPPV > NIV > NRBM. This assumption is based on the sequence of escalating ventilatory strategy in a clinical setting. However, as it was unclear, whether NIV or IPPV was a better ventilatory strategy at that point in time, the decision on ventilation was as per clinician discretion. Thus, patients were categorized as group 1 (IPPV - those patients who were intubated within 24 hrs of admission), group 2 (NIV - those patients who received NIV and without IPPV during the course of their stay in ICU), group 3 (NIV+ IPPV - those patients who received NIV initially and were intubated after 24 hours of admission) and group 4 (NRBM - those patients who received oxygen support by only Non-Rebreathing Mask). Charlson’s co-morbidity index, which assesses adult patients for over 30 co-morbid chronic conditions wherein each condition is weighed according to the algorithm and weights are summed for each patient to determine the final score and baseline Rox Index, the ratio of SpO_2_/FiO_2_ to respiratory rate were calculated for determining odds of mortality by multivariable logistic regression [16].

Statistical analysis: Statistical analysis was carried out by SPSS v23.0. The primary outcome of the study was all caused by in-hospital or 30-day mortality (whichever came first). The secondary outcome was the length of stay in the ICU, duration of hospitalization, and incidence of pneumothorax. The chi-square test was used to compare categorical variables and the ANOVA test to compare continuous variables. Odds of mortality were established by multivariable logistic regression. A P-value of ≤ 0.05 was considered statistically significant.

## Results

**Figure 1:**
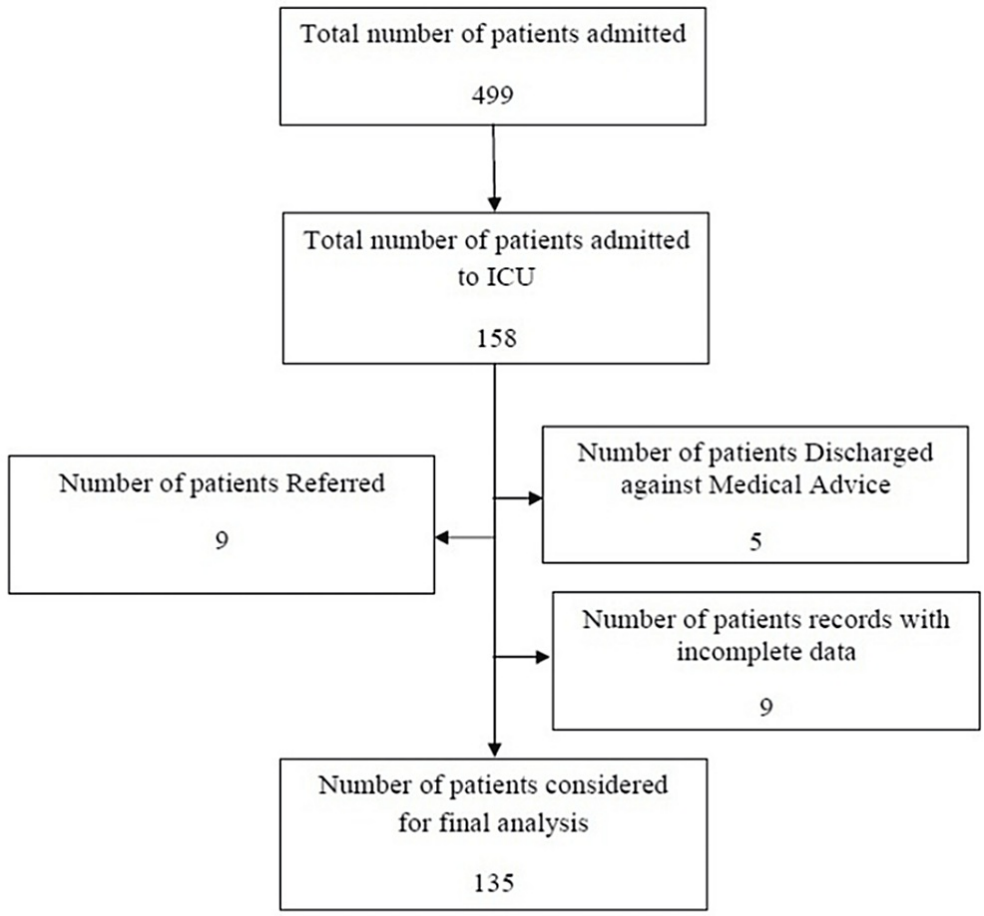
Flowchart

During the second wave in India, a total of 499 got admitted to our tertiary care center with a diagnosis of COVID-19. Of these, 158 patients were admitted to the critical care unit for ventilatory support or monitoring. Of these, 135 patients were considered for final analysis (Figure 1).

The baseline characteristics of the four groups are mentioned in Table 1.

**Table 1:** Baseline characteristics across the groups

SI No	Particular	Group-1 (IPPV) N =21	Group-2 (NIV) N=39	Group-3 (NIV+IPPV) N=32	Group-4 (NRBM) N=43	P-value
1	Age (mean)	61.09 ± 11.84	53.13 ± 14.06	49.34 ±14.73	53.30 ± 13.55	0.029
2	Gender	M=14, F=7	M=22, F=17	M=17, F=15	M=26, F= 17	0.77
3	Duration of symptoms at admission	5.62 ± 4.50	4.62 ± 2.73	5.50 ± 3.61	5.51 ± 4.88	0.69
4	Charlson’s Index	2.19 ±1.47	1.61 ± 1.54	1.21 ± 1.43	1.37 ± 1.23	0.08
5	Baseline SpO_2_ at admission	77.09 ± 24.62	87.95 ± 11.17	88.87 ± 8.27	91.23 ± 8.77	0.01
6	Rox Index	3.50 ± 2.53	4.47 ±2.02	4.50 ± 2.92	7.58 ± 6.48	0.00
7	SpO_2_/FiO_2_	1.00 ±0.62	1.20 ±0.50	1.27±0.69	1.82±1.30	0.001
8	LDH	539.50 ±364.17	561.41±227.19	602.81±260.83	376.54±171.03	0.002
9	CRP	9.08±6.71	24.78±48.05	9.41±6.74	9.87±6.83	0.05
10	S Ferritin	724.03±1736.78	1479.15±3588.23	493.90±504.59	516.71±610.88	0.431
11	Chest x-ray grade	3.13±1.95 (N=8)	4.78±1.53 (N=32)	4.24±1.73 (N=25)	4.17±1.96 (N=23)	0.11

The mean age in group 1 was significantly different between groups with a higher mean age of 61.09± 11.84. Post-hoc analysis showed a significant difference between groups 1 and 3, with group 3 having more younger age with a mean of 49.34 ± 14.73.

Duration of symptoms and Charlson’s index were comparable between the groups. Diabetes mellitus was the most common co-morbidity seen in those admitted to ICU. Baseline SpO_2_, Rox index, and SpO_2_/FiO_2_ ratio measured at admission were significantly different between groups. Post hoc analysis showed that groups 1, 2, and 3 had similar baseline Rox index and SpO_2_/FiO_2_ ratio; however, group 4 (only NRBM) had significantly higher values as compared to all other three groups (P ≤ 0.05).

The overall mortality rate among patients admitted at our center was 15% and the rate among patients admitted to the critical care unit was 54.07%. Among patients who required ventilatory support, patients who were intubated at first and those intubated following the trial of NIV had a significantly high mortality rate of 90.47% and 93.75%, respectively, as compared to 51.28% in patients who received only NIV. The number of days of hospitalization was significantly different between groups and post hoc analysis showed a significant difference between group 1 and group 2 (P=0.007). A similar finding was seen in a number of ICU days, with a significant difference between group 1 and group 2 in post hoc analysis (P=0.009). The overall incidence of pneumothorax was 10.37% with a significantly higher incidence in those intubated (22.64%) (Table 2).

**Table 2:** Outcome variables

SI No	Outcome		Group-1 (IPPV) N =21	Group-2 (NIV) N=39	Grou-3 (NIV+IPPV) N=32	Group-4 (NRBM) N=43	P-value
1	Primary outcome	Death	19	20	30	4	0.00
		Discharge	2	19	2	39	
2	Number of days of hospitalization		9.57±8.82	17.79±10.67	12.43±6.66	14.14±9.50	0.00
3	Number of ICU days		8.14±7.53	13.94±7.74	12.06±6.44	7.72±5.10	0.01
4	Pneumothorax		1	3	11	0	0.001

Multivariable regression analysis: In the initial analysis of baseline characteristics, as group 4 significantly differed on baseline SpO_2_, Rox index, and SpO_2_/FiO_2_ ratio regression analysis was done for group 1, group 2, and group 3. Based on available literature and univariate analysis, age, gender, Charlson’s index, baseline SpO_2_, Rox index, SpO_2_/FiO_2_, and group based on the ventilatory strategy adopted were considered for regression analysis [16-18]. The Omnibus test of model coefficients (P=0.00), and Hosmer and Lemshow test (P=0.214) showed regression model was statistically significant. The sensitivity of the model was 82.6% and the specificity was 52.2%. Group 3 and group 1 were associated with increased odds of mortality (Table 3).

**Table 3:** Odds ratio for mortality among patient’s characteristics and conditions

SI No	Variables	Odds ratio, 95% CI	P-value
1	NIV + IPPV	29.57 (4.51, 193.52)	0.001
2	IPPV	8.01 (1.35, 47.48)	0.02
3	Age	1.05 (0.95, 1.16)	0.32
4	Gender	0.51 (0.14, 1.82)	0.30
5	Charlson's Index	1.11 (0.44, 2.75)	0.83
6	Baseline SpO_2_	1.01 (0.96, 1.06)	0.71
7	Rox Index	0.52 (0.23, 1.19)	0.12
8	SpO_2_/FiO_2_	13.76 (0.42, 450.22)	0.14

## Discussion

This retrospective study was done to analyze the ventilatory strategy adopted in COVID-19 pneumonia patients admitted to ICU in our center during the second wave between March 2021 and August 2021. This analysis of 135 patients admitted to our ICU showed that the use of NIV was associated with improved chances of survival as compared to intubation without prior NIV. The odds of mortality were significantly high in those intubated first and in those who were intubated after the trial of NIV. 

In our study, 31.66% of hospitalized patients required admission to ICU. Among those considered for final analysis and admitted to ICU, 39.25% required invasive ventilation at some point during their ICU stay. The percentage observed is slightly higher as compared to the study by Wang et al. and Zirpe et al. [19]. Domingo et al. reported an increased requirement for ICU admission (17.5%) and an increased need for invasive mechanical ventilation (11.6%) during the second wave. Their study was based on the first and second wave in Spain which was between August and September 2020 [20]. This might be due to the severity of illness caused by COVID-19 strain (B.1.617.2 and B-.1.617.1) and also due to the type of patients getting admitted to hospital based on guidelines during second wave in India, wherein moderate to severe cases were recommended for admission, while mild cases were treated on OPD basis [2].

ARDS due to COVID-19 is different from ARDS caused by other etiological agents [21,22]. Use of NIV in type I respiratory failure and ARDS is still controversial [23,24]. However, its use in COVID-19 was supported by few studies as a useful mode of therapy [25]. Sivaloganathan et al. in their analysis of data from a single center observed 73% of patients had an initial trial of NIV and 27% underwent immediate tracheal intubation [26]. Bertania et al. in their interim analysis of the HOPE COVID-19 registry reported a survival of 56.6% in patients on NIV [25]. Forest et al. in their retrospective study had observed 83% mortality in patients on invasive ventilation as compared to 32% in patients on NIV with OR of 30 (95% CI 16-60) [27]. Our study finding is similar, with increased odds of mortality in patients with IPPV as compared to NIV. 

During the period of March 2021, when India saw a sudden rise in COVID-19 cases, with more moderate to severe cases getting admitted to hospital and hence ICU, the guidelines for a ventilatory strategy to be adopted were inconclusive. While some studies published at that point in time favored NIV, few reviews favored IPPV over NIV [4,28,29]. In our center, the decision to intubate a patient was based on the clinical condition of the patient, tolerance of NIV, availability of ventilator, and clinician's discretion. The most common indication for intubation in our center was refractory hypoxemia followed by not tolerating the NIV mask. The availability of helmet ventilators probably would have avoided intubation in a few patients and hence could have improved survival [30].

The mortality among those intubated within 24 hours of admission was higher as compared to those on NIV and was similar to those who had a trial of NIV before intubation. This is in contrast to the observation by Zirpe et al. wherein they observed significantly high mortality in those patients who underwent delayed intubation [19]. This difference might be due to the difference in the pathogenicity of strain involved in the first and second waves and also the difference in resources available in various intensive care settings. Daniel et al. in their secondary analysis found 66% decrease in the odds of mortality in patients intubated after a trial of NIV [31]. However, in our study, we did not observe any significant decrease in a secondary analysis.

This study is limited by sample size, a larger sample size including data from other centers might give more insights into the ventilatory strategy adopted in SARS-CoV-2. The data collected was based on the records in the patient file and the Hospital Management Information system (HMIS), which do not capture vital parameters and ventilatory data. Capturing such data in an electronic database and analysis of the same would probably give more clarity with regard to the cause of death in those intubated. It would help in understanding the changes in ventilatory mechanics as the disease progressed. Also, the increased mortality among intubated patients can be attributed to limited staff and resources available to manage a ventilated patient which could not be captured in our analysis.

## Conclusions

In a resource-limited setting, with overwhelming ICU during current pandemic, use of non-invasive ventilation is associated with higher survival in patients with COVID-19. The prognosis of patient who are intubated early or after trial of NIV is same with increased odds of mortality. Also, having a disaster management plan at the level of Institute might help in having a better outcome in the event of such a scenario in future.
